# Accurate Identification of Klebsiella variicola by MALDI-TOF Mass Spectrometry in Clinical Microbiology Laboratories

**DOI:** 10.1128/spectrum.02844-22

**Published:** 2022-09-20

**Authors:** Yuki Ohama, Yusuke Nomura, Miyuki Mizoguchi, Yoshimi Higurashi, Koh Okamoto, Sohei Harada

**Affiliations:** a Department of Infection Control and Prevention, The University of Tokyo Hospital, Tokyo, Japan; b Department of Infectious Diseases, The University of Tokyo Hospital, Tokyo, Japan; Johns Hopkins Hospital

**Keywords:** *Klebsiella pneumoniae*, *Klebsiella quasipneumoniae*, *Klebsiella variicola*, MALDI-TOF

## Abstract

Klebsiella variicola is a pathogen that is increasingly recognized as being associated with human infections, but the methods available to clinical microbiology laboratories for accurate identification are limited. In this study, we assessed the accuracy of identification of *K. variicola* by matrix-assisted laser desorption ionization–time of flight (MALDI-TOF) mass spectrometry using genetic identification with multiplex PCR as the reference method. Antimicrobial susceptibilities and virulence of *K. variicola* strains were also investigated. Fifty-five Klebsiella pneumoniae, 26 *K. variicola*, and 2 Klebsiella quasipneumoniae clinical strains were used for evaluation. Both MALDI Biotyper with library version 9 and Klebsiella MALDI TypeR, a web-based species identification tool using MALDI-TOF data, accurately identified all *K. variicola* strains. In addition, two strains of *K. quasipneumoniae* were accurately identified with Klebsiella MALDI TypeR. Whole-genome sequencing confirmed the accurate identification to the subspecies level by Klebsiella MALDI TypeR for four strains (two strains each of *K. variicola* subsp. *variicola* and *K. quasipneumoniae* subsp. *similipneumoniae*). While 13 strains, 3 strains, and 1 strain of K. pneumoniae showed nonsusceptibility to ampicillin-sulbactam, ceftriaxone, and meropenem, respectively, all strains of *K. variicola* were susceptible to all tested antimicrobial agents. Although two *K. variicola* strains were positive for the string test, no *K. variicola* strains harbored any of the genes associated with hypervirulence of K. pneumoniae. Accurate identification of the K. pneumoniae complex, including *K. variicola*, by MALDI-TOF in clinical microbiology laboratories is expected to clarify the clinical characteristics of each species in the future.

**IMPORTANCE** Recent widespread use of bacterial whole-genome sequencing analysis has resulted in the proposal of novel bacterial species and reclassification of taxonomy. Accurate methods for identification of bacterial species in clinical microbiology laboratories are essential to accumulate information on the clinical characteristics of each bacterial species. Klebsiella variicola is a member of the Klebsiella pneumoniae complex, and its association with human infections has been increasingly recognized, but accurate identification methods approved for use in clinical microbiology laboratories have been limited thus far. The findings of the present study suggest that *K. variicola* can be accurately identified by matrix-assisted laser desorption ionization–time of flight mass spectrometry using updated library or web-based identification tools. Accurate identification will promote exploration of clinical characteristics of *K. variicola*.

## OBSERVATION

Although Klebsiella variicola was first reported as a plant-associated bacterium; it was later shown to cause human infections (1, 2). It is difficult to differentiate *K. variicola* from other species of Klebsiella pneumoniae complex by biochemical identification methods or automated instruments. Although the inability to ferment adonitol was initially considered a characteristic of *K. variicola*, subsequent studies demonstrated that *K. variicola* and K. pneumoniae cannot be distinguished with certainty by this test (2, 3). Therefore, genetic identification by sequencing of the whole genome or the KVAR_0717 (*yggE*) gene is the most reliable method (4). Multiplex PCR targeting genes present only in each species of K. pneumoniae complex has also been proposed as a more widely available method (5, 6). While matrix-assisted laser desorption ionization–time of flight (MALDI-TOF) mass spectrometry has been increasingly used for bacterial identification in clinical microbiology laboratories in recent years, previous studies reported that misidentification of the species belonging to K. pneumoniae complex frequently occurs by this method (4, 7, 8).

In this study, the accuracy of identification of K. pneumoniae complex by MALDI-TOF with an updated library and with web-based species identification tool was evaluated. We used frozen-stored strains of K. pneumoniae (*n* = 52) and *K. variicola* (*n* = 16) recovered from blood culture and identified with a score of 2.00 or higher by MALDI Biotyper (Bruker Daltonics, Bremen, Germany) at the University of Tokyo Hospital (UTH) from January 2019 to July 2020. After the microbiology laboratory of UTH updated the library from version 4 to 9 in August 2020, 15 strains recovered from blood culture were identified as *K. variicola* with a score of 2.00 or higher until November 2021, and these strains were added for the analysis. Only one strain from each patient was used. K. pneumoniae FUJ00227 (accession no. JAMOBN000000000), Klebsiella quasipneumoniae subsp. *similipneumoniae* FUJ00228 (accession no. JAMOBM000000000), and *K. variicola* subsp. *variicola* FUJ01370 (accession no. GCA_019042755), bacterial species of which have previously been identified by whole-genome sequencing and >99% average nucleotide identity (ANI) with type strains using ANI Calculator (https://www.ezbiocloud.net/tools/ani), were added as reference strains (9).

Multiplex PCR for the unique genes of K. pneumoniae, *K. variicola*, and *K. quasipneumoniae* was performed as previously described (5). Fifty-five strains were identified as K. pneumoniae, 26 as *K. variicola*, and 2 as *K. quasipneumoniae*, and these results were used as the reference for later identification by MALDI-TOF. Reference strains were accurately identified by the multiplex PCR.

The study strains were retested with MALDI Biotyper with library version 9, and 57 were identified as K. pneumoniae and 26 as *K. variicola*. Although identification of all strains of *K. variicola* and K. pneumoniae was accurate, two *K. quasipneumoniae* strains were misidentified as K. pneumoniae with MALDI Biotyper with library version 9 (Table 1). Reference strains of K. pneumoniae and *K. variicola* were identified accurately, and that of *K. quasipneumoniae* was misidentified as K. pneumoniae. In the original identification with library version 4 at UTH, all *K. variicola* strains were identified accurately, but a minority of K. pneumoniae strains were misidentified as *K. variicola* (Table 1).

**TABLE 1:** Identification results for 83 strains obtained by each method^*a*^

Identification by MALDI	No. of strains identified by PCR^*b*^		
	K. pneumoniae (*n *= 55)	*K. variicola* (*n* = 26)	*K. quasipneumoniae* (*n* = 2)
MALDI Biotyper			
Library version 9			
K. pneumoniae	55	0	2
*K. variicola*	0	26	0
Library version 4^*a*^			
K. pneumoniae	51	0	1
*K. variicola*	3	12	1
Klebsiella MALDI TypeR			
K. pneumoniae	54	0	0
*K. variicola* subsp. *variicola*	0	26	0
*K. quasipneumoniae* subsp. *similipneumoniae*	0	0	2
K. pneumoniae complex	1	0	0

a The results for 68 strains in the microbiology laboratory at the University of Tokyo Hospital between January 2019 and July 2020 are presented (54 K. pneumoniae, 12 *K. variicola*, and 2 *K. quasipneumoniae* strains).

b Identification was performed using multiplex PCR (5).

Identification was also performed with Klebsiella MALDI TypeR (https://maldityper.pasteur.fr/) using MALDI-TOF data obtained by MALDI Biotyper (10). Fifty-four were identified as K. pneumoniae, 26 as *K. variicola* subsp. *variicola*, and two as *K. quasipneumoniae* subsp. *similipneumoniae*, and these results were accurate (Table 1). A strain (UTH00086) was reported as Klebsiella pneumoniae complex with no identifiable species. Reference strains were accurately identified.

UTH performs drug susceptibility testing using the MicroScan WalkAway system (Beckman Coulter, Brea, CA). The MICs were evaluated using CLSI M100-S27 (11). Although 13 strains, 3 strains, and 1 strain of K. pneumoniae showed nonsusceptibility to ampicillin-sulbactam, ceftriaxone, and meropenem, respectively, all strains of *K. variicola* and *K. quasipneumoniae* were susceptible to all antimicrobial agents (Table 2). Virulence of *K. variicola* strains were assessed with a string test and multiplex PCR identifying capsular genotype K1/K2 and major virulence genes (12). While two strains demonstrated a hypermucoviscosity phenotype with a string test forming a string of >5 mm, no strains had K1/K2 capsular genotypes, *rmpA*, or an aerobactin-associated gene (*iutA*), which were reported as biomarkers for hypervirulent K. pneumoniae (13).

**TABLE 2:** Antimicrobial susceptibilities of the strains

Antibiotic(s)	No. (%) of strains susceptible		
	K. pneumoniae (*n* = 55)	*K. variicola* (*n* = 26)	*K. quasipneumoniae* (*n* = 2)
Ampicillin-sulbactam	42 (76.4)	26 (100)	2 (100)
Piperacillin-tazobactam	52 (94.5)	26 (100)	2 (100)
Cefmetazole	55 (100)	26 (100)	2 (100)
Ceftriaxone	51 (92.7)	26 (100)	2 (100)
Ceftazidime	53 (96.4)	26 (100)	2 (100)
Cefepime	51 (92.7)	26 (100)	2 (100)
Meropenem	54 (98.2)	26 (100)	2 (100)
Levofloxacin	53 (96.4)	26 (100)	2 (100)
Gentamicin	51 (92.7)	26 (100)	2 (100)
Amikacin	55 (100)	26 (100)	2 (100)

Whole-genome sequencing of (i) strains with discordant bacterial identification results by MALDI-TOF and multiplex PCR and (ii) *K. variicola* strains with hypermucoviscosity was performed with Illumina MiSeq (Illumina, Inc., San Diego, CA) as described previously (14). Raw reads generated by MiSeq were quality trimmed with Trimmomatic tool (version 0.39) and assembled with SPAdes (version 3.15.4). Genetic characterization of the genomes was performed with software available at the Pathogenwatch website (https://pathogen.watch/). Species identified by whole-genome sequencing were identical to those identified by multiplex PCR (Table 3). Accurate identification of subspecies with Klebsiella MALDI TypeR for four strains was also confirmed. Among *K. variicola* strains, two strains exhibited hypermucoviscosity, but none harbored any of the genes associated with hypervirulence of K. pneumoniae (*rmpA*/*A2*, *ybt*, *clb*, and *iuc*) (13).

**TABLE 3:** Species identification results and sequence types of the strains for which whole-genome sequencing was performed^*a*^

Strain	MALDI Biotyper (Library version 9)	Klebsiella MALDI TypeR	Multiplex PCR	Whole genome sequencing	Sequence type
UTH00011	K. pneumoniae	**K. quasipneumoniae** subsp. *similipneumoniae*	**K. quasipneumoniae**	**K. quasipneumoniae** subsp. *similipneumoniae*	6080
UTH00079	K. pneumoniae	**K. quasipneumoniae** subsp. *similipneumoniae*	**K. quasipneumoniae**	**K. quasipneumoniae** subsp. *similipneumoniae*	334
UTH00086	**K. pneumoniae**	Not identified	**K. pneumoniae**	**K. pneumoniae**	35
UTH00026	**K. variicola**	**K. variicola** subsp. *variicola*	**K. variicola**	**K. variicola** subsp. *variicola*	6209 (319)
UTH00031	**K. variicola**	**K. variicola** subsp. *variicola*	**K. variicola**	**K. variicola** subsp. *variicola*	6235 (318)

a Whole-genome sequencing was performed because the strains showed discordant bacterial identification results by MALDI-TOF MS and multiplex PCR (UTH00011, UTH00079, and UTH00086) or were *K. variicola* with hypermucoviscosity (UTH00026 and UTH00031). Identification results consistent with multiplex PCR identification are shown in bold. Sequence types were determined according to the protocol described in BIGSdb-Pasteur databases (http://bigsdb.pasteur.fr/). Sequence types determined according to the protocol described in Klebsiella variicola MLST homepage (https://mlstkv.insp.mx/) are also indicated in parentheses for Klebsiella variicola isolates.

Recent studies showed that it is possible to identify species belonging to K. pneumoniae complex with MALDI-TOF by identifying the characteristic peak position (15, 16). MALDI Biotyper has increased the number of *K. variicola* strains included in the library—from 1 to 12—with the update to version 6. As a result, *K. variicola* was increasingly reported with high scores when the recent version of the library was used, but the accuracy of its identification has not been fully confirmed to date. This study demonstrated that the library update has enabled accurate identification of *K. variicola* by MALDI Biotyper. *K. quasipneumoniae* was not accurately identified by MALDI Biotyper, which was expected since data on the species are not included in the library.

This study also analyzed the accuracy of identification by Klebsiella MALDI TypeR (10). The results confirmed that *K. variicola* can be accurately identified and further suggest that *K. quasipneumoniae* and subspecies of K. pneumoniae complex may also be identified, although the number of verified strains was limited. Klebsiella MALDI TypeR can be used in a wide range of clinical laboratories using MALDI-TOF because it requires no additional specific equipment. In countries where the use of MALDI-TOF is not widespread, it is preferable that alternative methods for identification, including multiplex PCR, are available.

Fatal infections and multidrug-resistant strains of *K. variicola* have been reported (14, 17). Our analysis, together with the results of previous studies, suggests that there may be differences in clinical characteristics and drug susceptibility between K. pneumoniae and *K. variicola* even in the same geographic region, but more data are needed to draw definitive conclusions (18, 19). It is known that a certain proportion of strains of K. pneumoniae are hypervirulent and tend to cause severe infections (14). While two *K. variicola* strains had hypermucoviscosity, these strains harbored no genes associated with hypervirulence of K. pneumoniae. Although an IncFIB plasmid (pKV8917) that confers hypermucoviscosity to *K. variicola* in an *rmpA*-independent manner has been reported, plasmid analysis is beyond the scope of our study (20).

Recent studies have revealed the molecular characteristics of *K. variicola* (2, 21). Accurate identification using MALDI-TOF in clinical microbiology laboratories would lead to efficient collection of information on the clinical characteristics of this organism and would also aid in the collection of strains for molecular epidemiology studies.

### Data availability.

All genome sequences have been deposited in the NCBI database under BioProject accession no. PRJNA858671.
